# Salmon-IgM Functionalized-PLGA Nanosystem for Florfenicol Delivery as an Antimicrobial Strategy against *Piscirickettsia salmonis*

**DOI:** 10.3390/nano14201658

**Published:** 2024-10-16

**Authors:** Felipe Velásquez, Mateus Frazao, Arturo Diez, Felipe Villegas, Marcelo Álvarez-Bidwell, J. Andrés Rivas-Pardo, Eva Vallejos-Vidal, Felipe Reyes-López, Daniela Toro-Ascuy, Manuel Ahumada, Sebastián Reyes-Cerpa

**Affiliations:** 1 Centro de Genómica y Bioinformática, Facultad de Ciencias, Ingeniería y Tecnología, Universidad Mayor, Santiago 8580745, Chilemateus.frazao@mayor.cl (M.F.); arturo.diez@mayor.cl (A.D.); felipe.villegass@mayor.cl (F.V.); marcelo.alvarezb@mayor.cl (M.Á.-B.); jaime.rivas@umayor.cl (J.A.R.-P.); 2 Escuela de Biotecnología, Facultad de Ciencias, Ingeniería y Tecnología, Universidad Mayor, Santiago 8580745, Chile; 3 Centro de Biotecnología Acuícola, Universidad de Santiago de Chile, Santiago 9170002, Chile; eva.vallejosv@usach.cl (E.V.-V.); felipe.reyes.l@usach.cl (F.R.-L.); 4 Centro de Nanociencia y Nanotecnología CEDENNA, Universidad de Santiago de Chile, Santiago 9170002, Chile; 5 Núcleo de Investigaciones Aplicadas en Ciencias Veterinarias y Agronómicas, Facultad de Medicina Veterinaria y Agronomía, Universidad De Las Américas, La Florida, Santiago 8250122, Chile; 6 Laboratorio de Virología, Departamento de Biología, Facultad de Ciencias, Universidad de Chile, Santiago 8380000, Chile; danitoro@uchile.cl; 7 Centro de Nanotecnología Aplicada, Facultad de Ciencias, Ingeniería y Tecnología, Universidad Mayor, Santiago 8580745, Chile

**Keywords:** PLGA, nanosystem, florfenicol, *Piscirickettsia salmonis*, Atlantic salmon macrophages

## Abstract

Salmonid rickettsial septicemia (SRS), caused by *Piscirickettsia salmonis*, has been the most severe health concern for the Chilean salmon industry. The efforts to control *P. salmonis* infections have focused on using antibiotics and vaccines. However, infected salmonids exhibit limited responses to the treatments. Here, we developed a poly (D, L-lactide-glycolic acid) (PLGA)-nanosystem functionalized with Atlantic salmon IgM (PLGA-IgM) to specifically deliver florfenicol into infected cells. Polymeric nanoparticles (NPs) were prepared via the double emulsion solvent-evaporation method in the presence of florfenicol. Later, the PLGA-NPs were functionalized with Atlantic salmon IgM through carbodiimide chemistry. The nanosystem showed an average size of ~380–410 nm and a negative surface charge. Further, florfenicol encapsulation efficiency was close to 10%. We evaluated the internalization of the nanosystem and its impact on bacterial load in SHK-1 cells by using confocal microscopy and qPCR. The results suggest that stimulation with the nanosystem elicits a decrease in the bacterial load of *P. salmonis* when it infects Atlantic salmon macrophages. Overall, the IgM-functionalized PLGA-based nanosystem represents an alternative to the administration of antibiotics in salmon farming, complementing the delivery of antibiotics with the stimulation of the immune response of infected macrophages.

## 1. Introduction

Aquaculture represents an industry of significant economic importance as the primary source of animal protein in several countries and one of the most traded food commodities worldwide [1]. Aquaculture has experienced sustained growth since 1997, increasing from 34 million tons (Mt) to 87 Mt in 2020, and 94 Mt in 2022, representing 51% of total world fisheries and aquaculture (185 Mt) [2,3]. Regarding fishing, in 2020, farmed finfish reached 57.5 Mt, equivalent to USD 146.1 billion, including 49.1 Mt (USD 109.8 billion) from inland aquaculture and 8.3 Mt (USD 36.2 billion) from mariculture in the sea and coastal aquaculture on the shore [3]. However, aquaculture industries face numerous challenges, including meeting the demand, adapting to climate change, and ensuring biosecurity and disease control [4,5,6]. Regarding the latter, among the causes, infectious disease is the most relevant cause of death and industry challenges [7,8]. However, any action taken to meet the growing global demand for aquatic foods, its expansion, and future intensification must prioritize sustainability and benefits for the regions and communities most in need [3].

The aquaculture and salmon farming industry in Chile is essential to the country’s economy. It is the second largest worldwide salmon producer after Norway, with an associated income reaching USD 8827 million in 2022 [9]. The leading species farmed are Atlantic salmon (*Salmo salar*), with 65% of the total biomass produced during the first half of 2023, followed by coho salmon (*Oncorhynchus kisutch*), with 29%, and rainbow trout (*Oncorhynchus mykiss*) with 6% [10]. Nonetheless, the main problem of the salmon farming industry is the high prevalence of infectious diseases [10,11,12]. In Chile, the appearance of recurrent and aggressive outbreaks of Salmonid Rickettsial Syndrome (SRS) is the most severe health threat to the salmon industry [13,14,15,16]. *Piscirickettsia salmonis* is the etiological agent of SRS, a contagious systemic disease that mainly impacts salmon in saltwater [17]. *P. salmonis* is a Gram-negative bacterium, non-motile, unencapsulated, pleomorphic, and usually coccoid. Furthermore, it is described as a facultative intracellular pathogen that resides in vacuoles of macrophages and hepatocytes [17,18,19]. Economic losses due to infectious diseases, primarily caused by SRS, exceed USD 700 million annually, an amount that is rising alongside the increased use of antiparasitic and antibiotics [20].

The prophylaxis and control of *P. salmonis* have primarily centered on vaccines and antibiotics. However, neither strategy has been entirely effective [21]. Vaccines only provide short-term protection mediated by antibodies, which are ineffective against intracellular pathogens [20,21,22,23,24]. Antibiotics, mainly florfenicol and oxytetracycline, have been misused in fish farming to maintain high production rates. This excessive use occurs under conditions of high uncertainty and low effectiveness, leading to with a negative impact on marine ecosystems. Additionally, it promotes unwanted phenomena, such as bacterial resistance, posing a significant threat to public health globally [25]. On this line, bacterial resistance is recognized as an urgent and global issue. International agencies are currently adopting measures within the framework of “One Health”, an initiative that seeks to reduce antibiotic use in humans, animals, and farms while also limiting the spread of antibiotic resistance in the environment [26]. 

Despite this scenario, antimicrobials remain the gold standard for treating against SRS [22]. In Chile, according to a SERNAPESCA (Chile’s National Fisheries Service) report on the use of antimicrobials in national salmon fisheries, florfenicol (d-(threo)-1-(methylsulphonylphenyl)2-dichloroacetamide-3-floro-1-propanol) is the main antimicrobial used to treat SRS (90%) [27]. It is considered a derivative analog of chloramphenicol and thiamphenicol, non-volatile, with good tissue penetration in bacteria, and effective against Gram-positive and Gram-negative bacteria [28,29]. According to San Martin et al., a dose of 20 mg/kg l.w. administered orally through feed has a 100% probability of maximizing therapeutic success in infections caused by *P. salmonis* in salmonids [30]. However, the Veterinary Medical Registry of Chile recommends for various salmonid species a dose of 10 mg/kg l.w. [30]. Despite its wide use, in recent years, the toxic and side effects of florfenicol on broilers have been gradually reported. In poultry, a diet supplemented with 50 mg/kg of florfenicol inhibited embryo growth, affected normal heart development, and increased embryo mortality [31]. Moreover, overuse or misuse of florfenicol is known to promote the generation of antibiotic-resistant bacteria [32,33]. Thus, there is a need to develop new and more effective alternatives for treating infections, incorporating, for example, new advances related to molecular biology and technology at the nano scale.

Nanotechnology has allowed a reduction in the dosage of antimicrobials due to antibiotics bioavailability and pharmacokinetics improvements, bypassing certain limitations in clinical use [34]. The development of nanoparticles (NPs) capable of encapsulating drugs, such as antibiotics, to enhance drug delivery and release has been achieved by manipulating the NPs’ composition and size, resulting in a more effective and precise method of drug administration [35,36,37]. Among the different chemical reagents employed to prepare NPs, poly (D, L-lactide-glycolic acid) (PLGA) is an FDA-approved polymer that is currently widely used in the synthesis of nanoparticle systems proving be safe, biocompatible, and biodegradable. This polymer allows the formulation of particles with versatile physicochemical characteristics and degradation kinetics by varying the copolymer composition and molecular weight [38]. Moreover, PLGA has been extensively used to encapsulate and deliver different compounds in fish [39]. However, despite the biocompatibility of NPs, their efficacy is hampered by biological barriers, such as endothelial cells and mucosal barriers, which obstruct their targeting capabilities [40]. Conjugating nanoparticles with antibodies to improve efficiency could be a strategy to overcome these limitations [41]. In salmonids such as Atlantic salmon, antibody-mediated opsonization of particles enhances phagocytosis by macrophages through a mechanism potentially mediated by receptors similar to Fc receptors [42,43,44,45,46,47]. Previously, we demonstrated that latex-based non-specific IgM beads can induce lysosomal activation, confirming their capacity to reach the replicative niche of *P. salmonis* for an opsonized particle [48]. Now, we propose a new alternative for attending *P. salmonis* infection, where a PLGA-based nanoparticle carrying florfenicol is functionalized with salmon IgM targeting an effective antibiotic delivery. To this end, we characterize the PLGA-based nanoparticle carrying florfenicol through physicochemical assays, together with the encapsulation of florfenicol inside of the NP, while we measure their internalization capacity in salmon SHK-1 cells and the effect in infected SHK-1 cells. Finally, we discuss the potential impact of the proposed PLGA-based nanoparticles carrying florfenicol on intracellular infections in the aquaculture industry and their biological properties.

## 2. Materials and Methods

### 2.1. PLGA-Based NP Synthesis and Florfenicol Encapsulation

**Figure 1:**
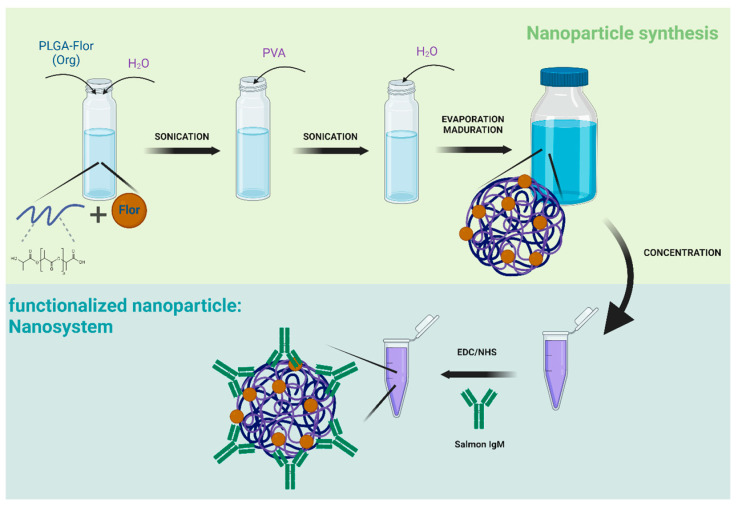
Schematic illustration of the nanosystem based on a PLGA-florfenicol nanoparticle conjugated with salmon IgM. The cartoon illustration was generated in BioRender.

The polymeric NPs were prepared by modifying the double emulsion (w1/o1/w2) solvent-evaporation method [49]. Briefly, 5 mg/mL of PLGA was dissolved in 2 mL of dichloromethane, and then 0.8 mL of MilliQ water was incorporated. The mixture was sonicated at 25% or 15% amplitude (150 watts) with a PULSE 150 Ultrasonic Homogenizer (Benchmark, Tempe, AZ, USA) with Probe 3 for 40 s. To the obtained mixture, 8 mL of 5 mg/mL polyvinyl alcohol (PVA) dissolved in MilliQ water was added and sonicated again at 25% or 15% amplitude for one minute. Then, 10 mL of MilliQ water was added, and the final mixture was left stirring for 16 h at 300 RPM to evaporate the dichloromethane. After evaporation, the NPs were matured with 30 mL of MilliQ water, stirring at 300 RPM for 24 h. Subsequently, the NPs were concentrated to 1 mL by centrifugation at 4000× *g* for 5 h using an Amicon^®^ Ultra 15 (Merck, Darmstadt, Germany), and both the NPs and their elution were stored at 4 °C (Figure 1). A similar protocol was employed to encapsulate the florfenicol in PLGA-based NPs, with the only difference being that 2 mL of the antibiotic was incorporated at a concentration of 10 mg/mL into the PLGA organic phase.

### 2.2. Salmon IgM Purification

The salmon IgM was extracted and purified using salmon blood, which Veterquímica S.A. kindly provided. The blood was centrifuged at 1500× *g* for 15 min at 4 °C to extract the salmon serum and was filtered through a 0.22 µm filter. Then, the IgM was extracted using a column with 2.5 mL of recombinant protein A-sepharose (Cytiva, Marlborough, MA, USA), previously equilibrated with 25 mL of binding solution (2.9 M NaCl and 0.1 M KCl). The serum was passed through the column and washed with approximately 30 mL of binding solution. The absence of residual serum protein was verified by measuring the absorbance at 595 nm using a Nanoquant Infinite M200 (TECAN, Grödig, Austria) spectrophotometer with 180 µL of Protein Assay Dye reagent and 20 µL of elution solution (0.1 M KCl). Posteriorly, the salmon IgM was eluted using the elution solution, generating 32 fractions of 500 mL each from the protein elution, with 75 µL of neutralization solution (1 M Tris, pH 11). The fractions were measured by a Bradford assay by spectrometry at 595 nm [50,51], and positive fractions were concentrated using an Amicon Ultra 4 (Merck) with a 30 kDa cutoff. SDS-PAGE verified the presence of salmon IgM and it was then stored at −20 °C. The maintenance and all procedures involving fish was performed in accordance with the ethical standards of the Institutional Bioethics and Biosafety Committee of the Universidad Mayor de Chile (approved in internal report No. 26/2022 dated 2 November 2022), and the relevant legislation in force.

### 2.3. IgM Conjugation to PLGA-Based NPs

The water content of a PLGA-based NP batch was removed by centrifugation at 4000× *g* for 20 min and posteriorly conjugated. The NPs were activated by adding 1 mL of a solution containing 19.5 mg/mL 2-morpholinoethanesulfonic acid monohydrate (MES), 12.5 mg/mL N-hydroxysuccinimide (NHS), and 5 mg/mL (1-ethyl-3-(3-dimethylamino) propyl carbodiimide (EDC) and constantly stirred at 4 °C. Subsequently, residual EDC-NHS was removed from the supernatant by centrifuging the nanoparticles at 4000× *g* for 20 min. Finally, 1 mL of Milli-Q water with 20 µg of salmon IgM was added, and the mixture was left to agitate constantly for 16 h at 4 °C. The conjugated nanoparticles were used immediately or lyophilized and stored at −20 °C (Figure 1).

### 2.4. Particle Size and ζ Potential Measurement

The size (average Z-size, nm) and surface charge (ζ potential, mV) of the prepared nanosystems were determined by dynamic light scattering and laser Doppler anemometry employing a Zetasizer Nano ZS (Malvern Instruments, Malvern, UK), respectively. Each preparation was suspended in 1 mL of phosphate-buffered saline (PBS; pH 7.4), and the size and ζ potential were calculated from 4 to 8 independent batches, respectively.

### 2.5. Florfenicol Encapsulation Efficiency Determination

The encapsulation efficiency of the PLGA-based NPs was obtained using a standard curve for florfenicol, generated through a serial dilution from 0 to 0.5 mg/mL. The absorbances were registered at 270 nm using a Jasco V-750 UV–Vis spectrophotometer (Tokyo, Japan) for the elution samples from the NPs with and without florfenicol. A correction was made to the obtained calibration curve and measured absorbance by incorporating the absorbance of the NPs without florfenicol (free PLGA), which allowed us to obtain the concentration of free florfenicol (*FF_free_*). Then, the florfenicol encapsulation efficiency was determined as the percentage (*EE*%) of the difference between the total (*FF_T_*) and *FF_free_*, as shown in Equation (1).

(1) $$EE\%=\left(\frac{{FF}_{T}-{FF}_{free}}{{FF}_{T}}\right)*100$$

### 2.6. SHK-1 Cell Line Culture

The SHK-1 cell line (*Salmo salar*, ECACC 97111106, European Collection of Authenticated Cell Cultures) was cultured at 16 °C in Leibovitz’s 15 medium (L-15, Hyclone, Logan, UT, USA) supplemented with 5% (*v*/*v*) fetal bovine serum (Hyclone), 4 mM L-glutamine (Corning, Corning, NY, USA), 1% (*v*/*v*) 2-mercaptoethanol (Gibco, Miami, FL, USA), 1× Penicillin/Streptomycin 100× (Corning), and 2.5 mg/mL Amphotericin B (Corning). For cell propagation, the monolayer was detached using 0.25% Trypsin EDTA (Corning) at a 1:10 ratio relative to the final bottle volume to be propagated according to the manufacturer’s instructions. The cell suspension was added to the supplemented L-15 medium, and trypsin was inactivated using 9 mL of the supplemented L-15 medium for every 1 mL of trypsin. Cells were seeded in cell culture bottles at 10,000 cells per cm^2^. Cells were observed daily under an AE-2000 inverted microscope (Motic, Fujian, China).

### 2.7. Confocal Microscopy

To visualize the nanosystem internalized in SHK-1 cells, 70,000 cells/well were incubated with approximately 50 nanosystems/cell for 3 h on top of coverslips of 12 × 12 mm. Then, the cells were fixed to the coverslips with 2% formaldehyde and incubated for 20 min. Basal cell fluorescence was eliminated using 150 mM ammonium chloride for 10 min. Further, cell permeabilization was carried out with 0.5% Triton X-100 for 15 min. Posteriorly, the cell cytoskeleton was labeled with phalloidin (1.5:100) for 20 min in darkness; the nucleus was stained with DAPI (1:5000) for 5 min, and, finally, the cells were blocked with 4% PBS-BSA and 0.1% Triton X-100 for 30 min. The nanosystem was detected with primary antibody hybridoma I-14 [52] (mouse IgG anti-salmon IgM), recognizing the constant region of salmon IgM and incubating them for 1 h on ice. Subsequently, the primary antibody was labeled with a secondary antibody, Alexa Fluor™ 488 goat anti-mouse IgG (Life Technologies, Carlsbad, CA, USA), incubating for 1 h. Finally, samples were mounted on glass slides with the addition of Fluoromount Aqueous Mounting Medium (Sigma-Aldrich, St. Louis, MO, USA). Cells were observed using a confocal microscope Axio Observer.Z1/7 (Zeiss, Jena, Germany), employing three channels: AF488-T1, DAPI-T2, and AF594-T2. Galleries were created with bidirectional scanning, considering 0.25 µm sections at a resolution of 1024 × 1024 pixels and using the objective lens Alpha Plan-Apochromat 63×/1.46 Oil Korr M27.

### 2.8. Bacterial Growth

Culture and propagation of *P. salmonis* (LF–89–like) were performed in salmonid cell line CHSE 214 (ATCC N°CRL-1682) as previously described by Fryer et al., 1992 [53] and used in our previous works [48,54,55]. The CHSE-214 cell line was maintained at 16 °C in minimal essential medium (MEM; Corning) supplemented with 10% (*v*/*v*) FBS (Hyclone), 10 mM HEPES buffer (Corning), and 1% (*v*/*v*) non-essential amino acids (Corning). The infection was observed through conventional inverted light microscopy (Motic AE31E, China) after 4 to 6 days post-infection (dpi) to determine the cytopathic effect on cells [56,57]. Bacteria were quantified using a Petroff–Hausser chamber (Hausser Scientific, Horsham, PA, USA) according to the instructions provided by the manufacturer.

### 2.9. Evaluation of the Cytotoxicity Induced by Nanosystem in SHK-1 Cells

To evaluate the effect of the nanosystem on cytotoxicity on SHK-1 cells, we quantified lactate dehydrogenase (LDH) released into the extracellular medium. The SHK-1 cells were seeded at 10,000 cells/well in 96-well flat bottom plates. Then, cells were incubated with nanosystem at a concentration equivalent to the delivery of 15 μg/mL of encapsulated florfenicol. As controls, an equivalent amount of nanoparticles conjugated to IgM but that did not contain florfenicol (IgM-NPs) and an equivalent amount of florfenicol (15 μg/mL) were used. Cytotoxicity was evaluated in SHK-1 cell culture at 3, 5, and 7 days post-incubation using the Pierce LDH Cytotoxicity Assay Kit (Thermo Scientific, Waltham, MA, USA), according to the manufacturer’s instructions. A positive cell death control (C+) is included in the LDH quantification kit and corresponds to detergents to break the plasma membrane whose release of LDH is equivalent to 100% of cell death. Negative control (C−) includes SHK-1 cells not subjected to treatments. Both values are considered when calculating the cytotoxicity determined for each experimental evaluation.

### 2.10. Gentamicin Protection Assay and Quantification of Intracellular Bacterial Load

A gentamicin protection assay was performed to recover the intracellular bacteria from the infected macrophage-like cells. Briefly, 3 × 10^5^ SHK-1 cells were seeded in 6-well flat-bottom plates. Cells were infected with *P. salmonis* at MOI 10 for 48 h and then treated with nanosystems, IgM-NPs, unencapsulated florfenicol, and non-treated for 24 h. The intracellular bacterium was recovered following the protocol described by Pérez-Stuardo et al., 2019 [54].

### 2.11. Detection of P. salmonis Using Quantitative Polymerase Chain Reaction (qPCR)

The gene encoding 16S rDNA (primers, Fw: 5′-AGG-GAG-ACT- GCC-GGT-GAT-A-3′; Rv: 5′-ACT-ACG-AGG-CGC-TTT- CTC-A-3′) was amplified as described by Karatas et al., 2008 [58]**,** to detect the presence of *P. salmonis* in the infected cell cultures, similarly to the method described previously in Perez-Stuardo et al., 2019 [54], and Perez-Stuardo et al., 2020 [48]. Genomic DNA was obtained using the Wizard™ Genomic DNA Purification kit (Promega, WI, USA) according to the instructions provided by the manufacturer. PCR amplification was performed using the PowerUp™ SYBR Green Master Mix (Thermo Scientific) following the manufacturer’s instructions. The primers were added to a final concentration of 0.4 µM, and 12 ng of total DNA. The qPCR was conducted on a QuantStudio 3 Real-Time PCR system (Thermo Scientific, Singapore). The quantification of 16S rDNA copies was performed through interpolation from the standard curve with the cycle threshold (Ct) value obtained for each sample. On the other hand, the amplification of 18S rDNA, which was used as an internal control gene, allowed to ensure that there were no effects associated with variations in cell number. The gene encoding 18S rDNA (primers, Fw: 5′-CCT-TAG-ATG-GGG-GCT-3′; Rv: 5′-CTC-GGC-GAA-GGG-TAG-ACA-3′) was amplified.

The results are expressed as a percentage of bacterial load (%) relative to the number of copies of 16S rDNA gene quantified in SHK-1 cells infected with *P. salmonis* without other treatments (only infection).

### 2.12. Software and Statistical Analysis

Statistical analyses were conducted using an unpaired *t*-test to compare two groups and a non-parametric one-way analysis of variance (ANOVA) test with a Dunn’s multiple comparison test. We used GraphPad Prism v8.02 software to calculate the mean value and to perform the statistical tests. All data were expressed as the mean ± standard error of the mean (SEM). The *p* values < 0.05 were accepted as significant. The acquired images were analyzed using FIJI (ImageJ) 2.14.0 [59].

## 3. Results

### 3.1. Physicochemical Properties of PLGA-Based Nanosystem

**Figure 2:**
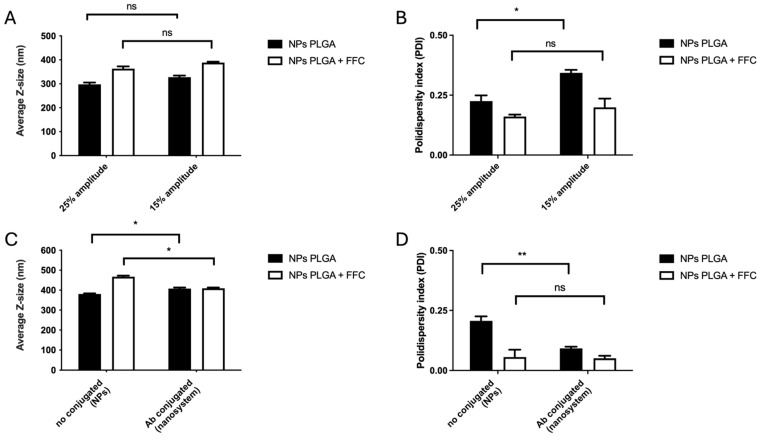
Characterization of the physicochemical properties of the nanosystem. (**A**) Z-size distribution and (**B**) PDI values based on amplitude percentage differences (25% and 15%) for sonication steps for PLGA NPs and PLGA NPs containing florfenicol (1 mg/mL). (**C**) Z-size distribution and (**D**) PDI values based on non-conjugated (PLGA-NPs) or Ab-conjugated (nanosystem) nanoparticles and PLGA NPs and PLGA NPs containing florfenicol (1 mg/mL). The statistical analysis was performed through an unpaired *t*-test. Values are given as mean ± standard error of the mean from three independent experiments. * *p* < 0.05; ** *p* < 0.01, ns: not significant.

In this work, one of the main goals was to produce uniform-size NPs that allow the characterization of the nanosystem properties. Thus, the effect of the amplitude during the sonication step on the size and distribution of the produced NPs was evaluated. The amplitude variations were assessed through DLS to measure the size (average Z-size) and distribution (PDI index). Using 25% or 15% of amplitude in each sonication step, we observed an inversely proportional change in the size of the NPs (Figure 2). For the batches at 25% amplitude, we obtained NPs with an average size of 300 nm, while for 15% amplitude, the size increased slightly to an average of 330 nm (Figure 2A, black columns). This result indicates that decreasing the amplitude from 25% to 15% for PLGA-based NP synthesis increases the nanoparticle size by approximately 10%. For each condition, the size distribution heterogeneity calculated from the polydispersity index (PDI index) indicates the absence of large aggregates (Figure 2B, black columns).

The next step was to evaluate whether the encapsulation of florfenicol affects the size of PLGA-based NPs. As florfenicol has low aqueous solubility [60], the antibiotic was incorporated in the organic phase along with PLGA. The amplitude conditions were kept for NP synthesis, as for PLGA-based NPs. A value of 25% or 15% of amplitude was employed in each sonication step and assessed through DLS to measure the size (average Z-size) and distribution (PDI index). It was not unexpected that lowering the sonication power from 25% or 15% in each sonication step, we obtained an inversely proportional change in the size for florfenicol-loaded NPs, which is consistent with our previous analysis from no-florfenicol PLGA-based NPs (Figure 2A, black columns). For florfenicol-loaded batches at 25% amplitude, we obtained an average size of 350 nm, while for 15% amplitude, the size increased slightly to an average close to 400 nm (Figure 2A, white columns). In addition, decreasing the amplitude from 25% to 15% for PLGA-based NP synthesis increases the nanoparticle size by approximately 14%. As in previous experiments, for each condition, the size distribution heterogeneity calculated from the polydispersity index (PDI index) indicates the absence of large aggregates (Figure 2B, white columns).

Once the synthesis of florfenicol-loaded PLGA-based NPs was established and their size characterized, the next step was to conjugate salmonid IgM to produce the fully functional nanosystem. Florfenicol-loaded PLGA-NPs were conjugated with Atlantic salmon IgM through carbodiimide chemistry, after previous activation of the NPs with the NHS/EDC couple (Figure 1). The size, polydispersity index, and ζ potential were assessed to characterize the effect of conjugation in the final nanosystem. For this, only 15% of amplitude was employed for these experiments. PLGA-based nanoparticles showed an average size of 380.5 nm compared to IgM-conjugated PLGA nanoparticles with an average size of 408 nm (Figure 2C, black columns). For both batches, the polydispersity index (PDI index) indicates the absence of large aggregates (Figure 2D, black columns). The ζ potential for each condition was −12.3 and −13.55 mV, indicating a slight difference in electronegativity of −1.25 mV between the PLGA-NPs compared to IgM-conjugated NPs (Table 1). Next, the effect of conjugation on florfenicol-loaded NPs was evaluated. The florfenicol-loaded PLGA-based nanoparticles exhibited an average size of 467.1 nm, whereas the nanosystem (IgM-conjugated florfenicol-loaded PLGA NPs) showed an average size of 409.4 nm (Figure 2C, white columns). In contrast to previous results, the nanosystem demonstrates a size reduction. Additionally, for both batches, the polydispersity index (PDI) indicates the absence of large aggregates (Figure 2D, white columns). The ζ potential for each condition was −10.87 and −9.96 mV, indicating the electronegativity surrounding PLGA by itself and IgM-conjugated NPs, respectively, with a slight difference of 0.9 mV observed in nanosystems of florfenicol-loaded nanoparticles (Table 1). These results indicate that the NPs, compared to the nanosystems, have a difference in ζ potential of 2.3 mV; however, the electronegativity remains unchanged.

### 3.2. Florfenicol Encapsulation Efficiency

To determine the capacity of PLGA nanoparticles to encapsulate florfenicol, we quantified the remaining concentration of florfenicol in solution post-synthesis via absorbance at 270 nm. A standard curve of florfenicol was constructed, and data were interpolated accordingly. Eight batches of NPs were synthesized using 2 mL of florfenicol (10 mg/mL), and 45 mL of preparation was collected. Then, to remove the NPs, the solution was filtered using a purification column with a 100 kDa cut-off, and eluted florfenicol was quantified by absorbance. As a result, the average encapsulation efficiency was 9.89% ± 1.42%.

### 3.3. IgM-NP Internalization in SHK-1 Cells

**Figure 3:**
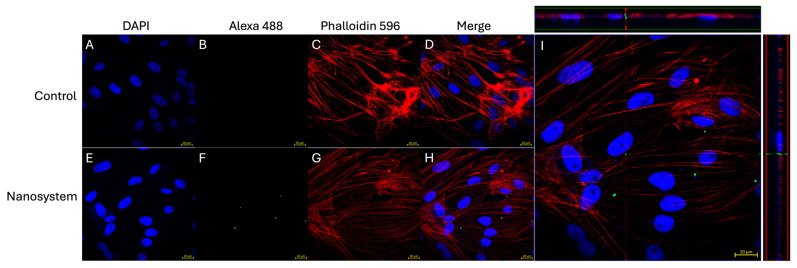
Intracellular detection of the nanosystem. SHK-1 cells were incubated with the nanosystem at 50 nanosystems/cell for 3 h. The cellular nucleus was stained with DAPI (blue), actin filaments to visualize the cell cytoplasm were stained with phalloidin (red), and Alexa 488 (green) was employed to visualize the IgM in the nanosystem. The intracellular location of the nanosystem was determined by a confocal microscope orthogonal image analysis of the z-stack obtained. (**A**–**D**) corresponds to images from non-stimulated SHK-1 cells (control) (scale bar 20 μm). (**E**–**H**) corresponds to images from SHK-1 cells incubated with the nanosystems (scale bar 20 μm). (**A**,**E**) nuclear stain (DAPI). (**B**,**F**) IgM detection on surface nanosystem by I-14 hybridoma and secondary antibody anti-mouse IgG Alexa 488. (**C**,**G**) Cytoskeleton detection by Phalloidin 596. (**D**,**H**) Merge of three fluorescent signals. (**I**) Orthogonal views of a midplane z section; height, 1.3 μm.

To determine whether the nanosystem can enter macrophage-like cells (SHK-1 cell line), we detected the Atlantic salmon IgM in the surface of functionalized PLGA-NPs within SHK-1 cells by confocal microscopy. The intracellular localization of the nanosystems was established by co-localization with specific cell structures. We used DAPI stain to identify the nucleus (blue), while using phalloidin for the actin filaments present in the cytoskeleton (red) (Figure 3). To specifically detect the Atlantic salmon IgM, we used a mouse IgG monoclonal antibody (hybridoma I-14) [52], which was recognized by an α-mouse IgG conjugated with Alexa488 (green).

Thus, SHK-1 cells were incubated with the nanosystem and imaged through confocal microscopy. Figure 3A,E shows the nuclear localization reported by DAPI stain and IgM detection was reported by Alexa488-conjugated α-mouse IgG, which is only observed in cells treated with the nanosystem (Figure 3B,F). Phalloidin stain highlights the actin filaments’ localization and the cytoplasm limits (Figure 3C,G). The merge of three fluorescent signals—blue, green, and red channels—is observed in Figure 3D,H. The simultaneous detection of IgM on the nanosystem surface, red-stained phalloidin (cytoplasm), and DAPI-stained nucleus on 1.75 μm thickness cross-sectional images by confocal microscopy showed that, after 3 h of incubation, nanosystem was found in the cytoplasm of macrophages-like cells. An orthogonal view of the cells (midplane z section; height: 2.00 μm) confirmed the intracellular localization of the nanosystem (Figure 3I).

### 3.4. Nanosystem Does Not Induce Cytotoxicity in SHK-1 Cells

**Figure 4:**
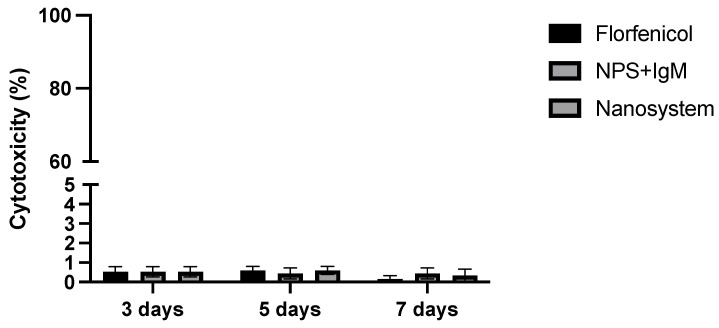
Evaluation of cytotoxicity induced by nanosystem. The cytotoxicity was evaluated at 3, 5, and 7 days post-incubation by the detection of LDH release into the extracellular medium. SHK-1 cells were incubated with nanosystem, IgM-NPs, or unencapsulated florfenicol (15 μg/mL). Values are given as the mean ± standard error of the mean from three independent experiments.

We used the LDH assay as a reporter for cell death to evaluate the cytotoxicity induced by nanosystem in SHK-1 cells at 3, 5, and 7 days post-incubation. As a result, we observed a minimal cytotoxicity in all treatments evaluated, close to 1% even after 7 days (Figure 4).

### 3.5. Effect of Nanosystems in Bacterial Load of P. salmonis When Infecting SHK-1 Cells

To evaluate the impact of the nanosystem on the intracellular bacterial load, we infected SHK-1 cells with *P. salmonis* at MOI 10 bacteria/cell for 48 h, and then cells were treated for 24 h with the nanosystems containing 15 μg/mL of florfenicol. An amount of 15 μg/mL of florfenicol and an equivalent number of IgM-NPs without florfenicol encapsulation were utilized as a control. Non-treated SHK-1 cells infected with *P. salmonis* incubated for 24 h in an L-15 medium were used as a positive infection control. The intracellular bacterial load was determined by quantitative RT-qPCR, and the results were reported as bacterial load percentage (%) relative to bacterial load detected in the positive control of infection (100% bacterial load).

**Figure 5:**
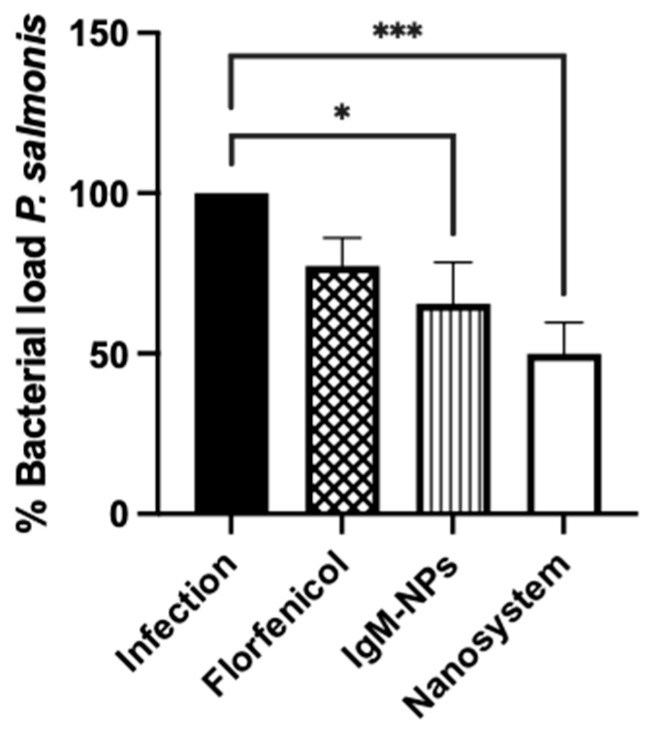
Quantification of intracellular *P. salmonis* bacterial load recovered from SHK-1 infected cells treated with nanosystem. SHK-1 cells were infected with *P. salmonis* MOI 10 bacteria/cell for 48 h. Then, cells were treated for 24 h with the nanosystem, 15 μg/mL of unencapsulated florfenicol (Florfenicol), or an equivalent number of PLGA NPs functionalized with IgM but without florfenicol (IgM-NPs). In the positive infection control, SHK-1 cells were infected for 48 h and maintained without other treatments for 24 h. The bacterial load was determined by quantification of 16S rDNA copies/cell by qPCR. The statistical analysis was performed through non-parametric ANOVA with Dunn’s multiple comparison test. Values are given as the mean ± standard error of the mean from three independent experiments. *: *p* < 0.05; ***: *p* < 0.001.

The effect of the nanosystem on bacterial load is reduced by more than 50% (Figure 5, white bar) compared to the *P. salmonis* infection control (Figure 5, black bar). As a control, SHK-1 infected cells were incubated with florfenicol 15 μg/mL (Figure 5, bar with diamond pattern), corresponding to an equivalent florfenicol concentration delivered by the nanosystem, where bacterial load was reduced to 77% of that detected in the positive infection control. Finally, PLGA NPs functionalized with IgM, but in the absence of florfenicol (Figure 5, bar with vertical line pattern), reduced the bacterial load to 65% of that detected in the positive infection control.

## 4. Discussion

Nanoparticles represent one of the main drug delivery systems suitable for most administration routes. These can act as potential carriers for several drugs, such as anticancer agents, antihypertensive agents, immunomodulators, hormones, and macromolecules, such as nucleic acids, proteins, peptides, and antibodies [61]. In this regard, nanomaterials can be designed for site-specific drug delivery. However, the targeting capability of nanoparticles is influenced by parameters such as particle size, surface charge, surface modification, and hydrophobicity [61].

To produce the nanosystem, the florfenicol-loaded PLGA NPs were first prepared using a modification of the double emulsion solvent-evaporation method. A relevant aspect during the synthesis is the NPs’ size, a crucial feature determining the treatment performance because it influences circulating half-life, cellular uptake, and drug release kinetics [53]. Previous work determined NPs smaller than 3 μm are more efficient in attachment and phagocytosis [54], which is desirable to take advantage of. In this work, florfenicol-loaded NPs have a higher size than empty NPs, which has been reported in other nanoparticles that contain drugs. For instance, for epirubicin loaded in PHB or PHBV nanoparticles [62,63] and roxithromycin-loaded PLGA nanoparticles [64], the size increases with respect to empty NPs, where it has been suggested that drug incorporation into the formulation caused the expansion of polymeric matrix resulting in an increased size of nanoparticle [62,64]. Further, the colloidal surface charge of the NPs was evaluated through ζ potential measurements. The absolute ζ value is commonly used as a colloidal stability parameter, associated with higher stability when the value moves away from 0 [65]. However, it must be considered that ζ potential is not a direct measure of the stability but the electric surface potential of the NPs, which allows an approximation to the colloidal stability [66]. The obtained results for the different formulations are closely related with a ζ potential close to −10 mV, indicating medium stability, which is correlated with the average sizes obtained. Their slight modifications regarding summative charges and molecular/packaging reorganization can be explained when preparing the different NPs [67].

The florfenicol-loaded PLGA-NPs were conjugated with Atlantic salmon IgM through carbodiimide chemistry, previous activation of the NPs with the NHS/EDC couple (Figure 1). This process promotes covalent bonds between PLGA-NPs and the IgM Fab region, exposing the Fc region. Conjugating antibodies to NPs enhances their delivery efficiency, enabling targeted tissue delivery and interaction with specific molecules in diverse organisms [41]. Carbodiimide chemistry is a commonly employed method for antibody conjugation to NPs. However, this process, which utilizes carboxyl groups on nanoparticle surfaces and amino groups on antibodies, has a drawback. While carbodiimide chemistry facilitates antibody binding to NPs via the Fab region, which provides specificity, it also hides this crucial region, rendering the antibody ineffective [68,69]. However, this drawback presents an opportunity in our model. Carbodiimide chemistry promotes antibody binding to PLGA NPs via the Fab region, exposing the Fc region to conjugated NPs and simulating an immune complex. In immune system cells like macrophages, after antigen recognition and immune complex formation, the exposed Fc region of an antibody interacts with the complement system to induce complement-dependent cytotoxicity or with the Fc receptors on natural killer (NK) cells or macrophages to induce antibody-dependent cellular cytotoxicity (ADCC) or antibody-dependent cellular phagocytosis (ADCP) and subsequent nanoparticle entry [70,71].

Passive immunization strategies are extensively used against various pathogens to stimulate the endocytic pathway through the Fc–FcR interaction. This interaction triggers effector mechanisms that increase phagocytosis and cytokine release. Notably, the Fc–FcR interaction plays a key role in directing vesicular trafficking, which is often disrupted by intracellular pathogens, thereby facilitating the lysosomal degradation of bacteria [48,72,73,74,75,76]. Joller et al. reported that IgG-functionalized latex microparticles were used to treat *Legionella pneumophila* infections in a mouse macrophage-like cell line RAW264.7. The authors suggested that the Fc region exposed from microparticles interacts with FcR in macrophages, preventing the host cell from replicating the pathogen and directing it to the lysosomes. This process depends on the kinases involved in FcR signaling [76]. Similarly, Pérez-Stuardo et al. evaluated the effect of IgM-functionalized latex microparticles in Atlantic salmon macrophages infected by *P. salmonis*, which promotes the lysosomal activity by lowering lysosomal pH, increasing the lysosomal proteolytic activity, and reducing the bacterial load [48].

Antibiotic-encapsulating nanoparticles have recently been described as a promising strategy for controlling and targeting the release of antibiotics directly to tissues infected with intracellular pathogens [77,78,79,80,81,82]. Toti et al. conducted a study in which the antimicrobial capacity of rifampicin- and azithromycin-encapsulating PLGA nanoparticles was evaluated against *Chlamydia trachomatis* and *Chlamydia pneumoniae* infections in the McCoy and HEp2 mouse cell lines. They observed that the treatment with free antibiotics failed to significantly reduce the number of intracellular inclusions where the bacteria were found; however, treatment with encapsulating nanoparticles significantly improved the effectiveness of both antibiotics [78]. Similarly, Pillai et al. conducted a study that synthesized nafcillin-loaded PLGA nanoparticles and evaluated their antimicrobial effect against *Staphylococcus aureus* infections in a cell culture of neonatal mouse osteoblasts. The results obtained by Pillai et al. demonstrate a significant decrease in nafcillin-loaded nanoparticle treatments compared to the positive infection control and treatments with nanoparticles without antibiotics, highlighting the potential of antibiotic-encapsulating nanoparticles as a treatment against intracellular pathogens [77]. Jiang et al. evaluated the antimicrobial capacity of gentamicin-encapsulated PLGA nanoparticles against *Klebsiella pneumoniae* infections in a murine model using the macrophage-like MH-S cell line. The antimicrobial capacity was estimated by colony-forming units (CFUs). They observed that treatment with florfenicol-loaded nanoparticles significantly reduced the colony-forming units compared to the free antibiotic and nanoparticle-free controls [82]. Dimer et al. reported similar results using clarithromycin-encapsulated PLGA nanoparticles to decrease the bacterial load of *S. aureus* infections in the mouse macrophage-like cell line RAW264.7. Treatment with clarithromycin-loaded nanoparticles reduced the intracellular bacterial load 1000-fold more than treatment with free antibiotics [79]. The results presented here are consistent with the studies mentioned above. The nanosystem treatment was the most successful, significantly reducing the bacterial load by more than 50%, being more effective than the unencapsulated florfenicol treatment at an equivalent concentration. It should be noted that this reduction in bacterial load is not due to a cytotoxic effect on SHK-1 cells that could explain the lower bacterial load detected. SHK-1 cells have been incubated for up to 7 days with the nanosystem, with IgM-NPs, and with florfenicol (15 μg/mL), and none of these treatments has a cytotoxic effect on Atlantic salmon macrophages (SHK-1 cells). Although more experiments are required to obtain more conclusive results, the results obtained are a good indication of the potential use of florfenicol-loaded PLGA nanoparticles against infectious outbreaks of *P. salmonis* and, thus, for reducing the excessive use of antibiotics by Chilean salmon farming today.

## 5. Conclusions

In global aquaculture, one of the main challenges is to reduce mortality due to infectious diseases, which allows maximizing food production [7,8]. For this purpose, it is of great importance to explore new strategies for drug delivery and increase the efficiency of the molecules used. This idea takes relevance when therapies should aim to fight and control intracellular pathogens, where traditional methods fail or display low efficiency. Thus, the development of new and different organic compatible polymers that allow an adequate delivery of natural bioactive agents [83] or even another type of nanodevices that allows efficient delivery of antibiotics or vaccines, including hybrid nanoparticles [84], micro/nanorobots to deliver poorly permeable macromolecules [85,86,87], or even novel antibacterial agents, such as nanoantibiotics [88], could have an impact on the current aquaculture.

In the current study, we developed an IgM-functionalized PLGA-based nanosystem to exploit the ability to activate the endocytic pathway of Atlantic salmon macrophages through Fc–FcR interaction, as previously we reported in Pérez-Stuardo et al., 2020 [48]. Moreover, the nanosystem is loaded with florfenicol, ensuring a local and specific antibiotic delivery in macrophages infected by *P. salmonis*. Thus, our nanosystem could be used as a potential alternative for antimicrobial strategy against *P. salmonis* infection. However, long-term treatments are needed if this technology is planned for salmon farming.

## Figures and Tables

**Table 1:** Average Z-size (hydrodynamic size, nm), polydispersity index (PDI), and ζ potential (mV) for each formulation of NPs based on PLGA.

Formulation	Average Z-Size (nm)	PDI	ζ-Potential (mV)
PLGA (nanoparticles)	380.5 ± 5.9	0.21 ± 0.032	−12.3 ± 0.14
PLGA conjugated	408.0 ± 9.5	0.09 ± 0.012	−13.55 ± 0.32
PLGA florfenicol	467.1 ± 9.7	0.07 ± 0.07	−10.87 ± 0.20
PLGA florfenicol conjugated (nanosystem)	409.4 ± 6.6	0.04 ± 0.027	−9.96 ± 0.29

## Data Availability

Data are contained within the article.
